# Type I interferon in patients with systemic autoimmune rheumatic disease is associated with haematological abnormalities and specific autoantibody profiles

**DOI:** 10.1186/s13075-019-1929-4

**Published:** 2019-06-14

**Authors:** John A. Reynolds, Tracy A. Briggs, Gillian I. Rice, Sathya Darmalinggam, Vincent Bondet, Ellen Bruce, Mumtaz Khan, Sahena Haque, Hector Chinoy, Ariane L. Herrick, Eoghan M. McCarthy, Leo Zeef, Andrew Hayes, Darragh Duffy, Ben Parker, Ian N. Bruce

**Affiliations:** 1 Centre for Musculoskeletal Research, Division of Musculoskeletal and Dermatological Sciences, School of Biological Sciences, University of Manchester, Manchester, UK; 2 NIHR Manchester Biomedical Research Centre, Manchester University NHS Foundation Trust, Manchester Academic Health Science Centre, The University of Manchester, Manchester, UK; 3 Manchester Centre for Genomic Medicine, St Mary’s Hospital, Manchester University NHS Foundation Trust, Manchester Academic Health Sciences Centre, Manchester, UK; 4 Division of Evolution and Genomic Sciences, School of Biological Sciences, University of Manchester, Manchester, UK; 5 Immunobiology of Dendritic Cells, Institut Pasteur, Paris, France; 6 INSERM, UMRS-1223, 75015 Paris, France; 7 Wythenshawe Hospital, Manchester University NHS Foundation Trust, Manchester Academic Health Science Centre, The University of Manchester, Manchester, UK; 8 Rheumatology Department, Salford Royal NHS Foundation Trust, Manchester Academic Health Science Centre, Salford, UK; 9 Bioinformatics Core Facility, Faculty of Biology, Medicine & Health, University of Manchester, Oxford Road, Manchester, UK

**Keywords:** Systemic autoimmune rheumatic disease, Interferon alpha, Autoantibodies

## Abstract

**Objectives:**

To investigate the relationships between interferon alpha (IFNα) and the clinical and serological phenotype of patients with systemic autoimmune rheumatic disease (SARDs) in order to determine whether a distinct subpopulation of patients can be identified.

**Methods:**

We recruited patients with at least 1 SARD clinical feature and at least 1 SARD-related autoantibody from two NHS Trusts in Greater Manchester. A 6-gene interferon-stimulated gene (ISG) score was calculated in all patients, and in a subgroup, a 30-gene ISG score was produced using NanoString. A digital Single Molecule Array (Simoa) was used to measure plasma IFNα protein. In an exploratory analysis, whole blood RNA sequencing was conducted in 12 patients followed by RT-qPCR confirmation of expression of 6 nucleic acid receptors (NARs) in the whole cohort.

**Results:**

Sixty three of 164 (38%) patients had a positive ISG score. The 3 measures of IFNα all correlated strongly with each other (*p* < 0.0001). There were no differences in mucocutaneous or internal organ involvement between the ISG subgroups. The ISG-positive group had increased frequency of specific autoantibodies and haematological abnormalities which remained significant after adjusting for the SARD subtype. Expression of DDX58, MB21D1 and TLR7 was correlated with the ISG score whilst TLR3, TLR9 and MB21D1 were associated with neutrophil count.

**Conclusion:**

In SARD patients, IFNα-positivity was associated with specific autoantibodies and haematological parameters but not with other clinical features. The variable NAR expression suggests that different pathways may drive IFNα production in individual patients. The identification of an IFNα-positive subgroup within a mixed SARD cohort supports a pathology-based approach to treatment.

**Electronic supplementary material:**

The online version of this article (10.1186/s13075-019-1929-4) contains supplementary material, which is available to authorized users.

## Introduction

Systemic Autoimmune Rheumatic Diseases (SARDs) are a group of multisystem autoimmune disorders with overlapping clinical and serological manifestations. Patients may transition between clinical disease categories over time; some evolve from an undifferentiated connective tissue disease (UCTD) to a specific disease type, whilst others develop an overlap syndrome. The significant commonality in clinical features and autoantibody profiles between some conditions suggests a shared molecular aetiology, whilst within a single-disease group significant molecular heterogeneity has been described [1]. Studies seeking to understand disease pathogenesis, identify reliable biomarkers, or develop novel treatment strategies may therefore be improved if undertaken in a molecularly defined subgroup of SARD patients, rather than in traditionally classified individual conditions [2]. The molecular taxonomy of SARDs offers new opportunities in clinical trial design; therefore, it is essential to better understand whether true immunopathological subsets of patients exists within clinical SARD cohorts.

Previous single-disease studies have demonstrated an increase in type I interferon (IFN) in some SARDs (notably Systemic Lupus Erythematosus (SLE) and Sjogren’s syndrome (SS)) [3–5]. However, it is less clear whether those SARD patients expressing a high level of IFNα represent a homogenous molecular subgroup.

The type I IFNs are a family of 17 cytokines compromising predominantly of IFNα subtypes. Thirteen IFNα subtypes have been identified in humans, with the coding genes all located on chromosome 9 [6]. Studies to date have been challenging due to the relatively low abundance of IFNα in the circulation, and the lack of adequately specific antibodies to confidently distinguish between the IFNα subtypes and between IFNα and other type I and type III IFNs (IFNλ). The ‘gold standard’ has therefore been the measurement of interferon-stimulated genes (ISGs) either directly in human samples or via an ex vivo stimulation assay [7, 8]. More recently, a highly sensitive digital ELISA has been trialled in adult and paediatric patients with SLE and juvenile dermatomyositis [9, 10].

We aimed to investigate type I IFN pathway activity using multiple methodologies in an unselected adult SARD cohort and to determine how this related to the clinical and immunological phenotype of patients. We also used unbiased transcriptome analysis of a subgroup of patients and investigated relevant IFN pathway molecules in order to move beyond classical physician diagnoses and towards a molecular taxonomy of SARDs.

## Methods

### Patients and healthy volunteers

Patients were recruited into the Lupus Extended Autoimmune Phenotype (LEAP) cohort from Manchester University NHS Foundation Trust (3 sites) and Salford Royal NHS Foundation Trust (1 site). The LEAP cohort includes patients with SLE, SS, UCTD, mixed connective tissue disease (MCTD), systemic sclerosis (SSc) and idiopathic inflammatory myopathies (IIM). Ethical approval was obtained from the Greater Manchester East Research Ethics Committee (13/NW/0564), and the study was conducted in accordance with the Declaration of Helsinki. Patients with an established diagnosis and clinically stable disease were eligible for inclusion if they had ≥ 1 clinical feature of a connective tissue disease and ≥ 1 relevant autoantibody. Autoantibodies were measured according to physician discretion but all included the BioPlex 2200 ANA Screen with MDSS (comprising the following autoantigens: dsDNA, Chromatin/nucelosome, Ro/SS-A, La/SS-B, Smith [Sm], SmRNP, RNP, Scl-70, Jo-1 and centromere). The physician (rheumatologist) diagnosis was used as the primary classifier of patients. Detailed clinical and serological data were recorded. Whole blood was collected for subsequent RNA analysis. Routine clinical biochemical and haematological parameters were measured, and additional plasma samples were collected for IFNα protein measurement (see below).

Healthy volunteers were recruited to provide comparator cohorts. For measurement of the ISG score, 29 healthy controls (16/29, 55% female), with a median age 24 years, were recruited (this cohort has been as previously described [11]). For the NanoString analysis, 27 of the 29 volunteers above were sampled: 16/27 (59%) female, median age 34 years. For the nucleic acid receptors analysis, 16 healthy controls (14/16 (88%) female) with a median age of 42 years were used.

### ISGs

Blood was collected into PAXgene tubes (PreAnalytix) kept at room temperature for between 2 and 72 h, frozen at − 20 °C, then stored at − 80 °C until extraction. Total RNA was extracted from whole blood using a PAXgene (PreAnalytix) RNA isolation kit. RNA concentration was assessed by Qubit 2.0 fluorometer (Thermo Fisher Scientific). After DNAse treatment, RNA was converted into cDNA with the high-capacity cDNA reverse transcriptase kit (Qiagen). Quantitative reverse transcription polymerase chain reaction (RT-qPCR) analysis was performed using the TaqMan Universal PCR Master Mix (Applied Biosystems), and cDNA derived from 40 ng total RNA. Using TaqMan probes for *IFI27* (Hs01086370_m1), *IFI44L* (Hs00199115_m1), *IFIT1* (Hs00356631_g1), *ISG15* (Hs00192713_m1), *RSAD2* (Hs01057264_m1) and *SIGLEC1* (Hs00988063_m1), the relative abundance of each target transcript was normalised to the expression level of *HPRT1* (Hs03929096_g1) and *18S* (Hs999999001_s1) and assessed with the Applied Biosystems StepOne Software v2.1 and Data Assist Software v.3.01. For each of the six probes, individual (patient and control) data were expressed relative to a single calibrator. The median fold change of the six ISGs, when compared to the median of the combined data of healthy controls, was used to create an interferon score for each patient as previously described [11]. RQ is equal to 2^−ΔΔCt^, i.e. the normalised fold change relative to a control. The mean interferon score of the healthy controls plus two standard deviations above the mean (+ 2SD) was calculated. Scores above this value (> 2·466) were designated as positive.

### Nucleic acid receptors (NARs)

RNA was extracted from whole blood, and RT-qPCR analysis was performed as described above using *18S* and *HPRT1* as reference probes. TaqMan probes for *DDX58* (Hs01061436_m1), *MB21D1* (Hs00403553_m1), *TMEM173* (Hs00736956_m1), *TLR3* (Hs01551079_g1), *TLR7* (Hs01933259_s1) and *TLR9* (Hs00370913_s1) were used. For each of the six probes, individual (patient and control) data were expressed relative to a single calibrator.

### Quantification of interferon alpha (IFNα) protein levels in plasma using a single molecule array (Simoa)

As described by Rodero et al., the Simoa IFNα assay was developed using a Quanterix Homebrew Simoa assay according to the manufacturer’s instructions and utilising two autoantibodies specific for IFNα isolated and cloned from 2 APS1/APECED patients [9, 12]. The 8H1 antibody clone was used as a capture antibody after coating on paramagnetic beads (0.3 mg/mL), and the 12H5 was biotinylated (biotin/Ab ratio = 30/1) and used as the detector. Recombinant IFNα17/αI (PBL Assay Science) was used as a standard curve after cross-reactivity testing. The limits of detection (LOD) were calculated by the mean value of all blank runs + 3SDs and was 0.23 fg/ml.

### NanoString ISG analysis

Gene expression of 30 interferon-stimulated genes and 3 housekeeping genes (HPRT1, NRDC, OTUD5, for details see Additional file 1) was measured. A 30 gene score was calculated based on the same methods for the RT-qPCR score (+ 2SD of healthy controls). Scores above 1.642 were considered positive. All ISG, NAR and NanoString measurements were conducted on single RNA samples.

### Gene expression analysis by RNA sequencing (RNA-Seq)

Whole transcriptome expression analysis was performed using samples from 12 participants, selected according to the ISG score and presence/absence of anti-Smith antibodies (see Additional file 1: Supplementary methods for details). Gene lists were uploaded into Ingenuity Pathway Analysis (http://www.ingenuity.com) in order to determine differentially regulated canonical pathways in the patients. ISGs were identified within the RNA-Seq dataset by comparing differentially expressed genes with the Interferome v2.0 database [13] (accessed from http://www.interferome.org/interferome/home.jspx on 8/6/2018).

### Availability of data

The RNA-Seq dataset supporting the conclusions of this article is available in the Array Express repository, (E-MTAB-7080, https://www.ebi.ac.uk/arrayexpress/experiments/E-MTAB-7080).

### Statistical analysis

Statistics were calculated using STATA v.13 (STATcorp, USA), GraphPad Prism version 5.0d for Mac OS X and version 7.00 for Windows (San Diego, CA, USA) and R v3.4.1. Non-parametric statistical tests were used (Mann-Whitney *U* and Kruskal-Wallis for comparison of 2 or > 2 groups, respectively, Spearman *r* for correlations) unless specified otherwise. Univariate linear and logistic regression models were used. Multivariable models all included age, gender and ethnicity plus additional relevant variables as described.

## Results

### ISG expression in patients with SARDs

We recruited a total of 164 patients with a median (IQR) age of 48.5 (36.8, 57.3) years and disease duration of 7.11 (3.16, 15.8) years (Table 1). The majority (122/163, 74.8%) of patients were Caucasian reflecting a clinical population in North West England and 155 (94.5%) were female. The most prevalent conditions in our cohort were SLE (67 patients, 40.9%) and UCTD (43 patients, 26.2%) (Table 1).

**Table 1:** Demographic characteristics of the patient population

	Whole cohort (*n* = 164)	ISG negative (*n* = 101)	ISG positive (*n* = 63)	*p* value (ISG positive vs. negative)
Age (years)	48.5 (36.8, 57.3)	50.3 (40.6, 59.5)	44.8 (31.6, 52.8)	0.012
Gender, female	155 (94.5)	97 (96.0)	58 (92.1)	0.277
Disease duration (years) *n* = 158	7.11 (3.16, 15.8)	6.84 (3.06, 15.3)	8.08 (3.56, 16.1)	0.538
Age at disease onset (years)	36.5 (26.2, 47.4)	40.5 (30.1, 48.4)	31.6 (23.6, 41.1)	0.005
Ethnicity				
Caucasian	122 (74.8)	80 (79.2)	42 (66.7)	0.430
Mixed	1 (0.6)	1 (0.99)	0	
Asian or Asian British	8 (4.9)	3 (2.97)	5 (7.94)	
Black or Black British	24 (14.6)	12 (11.9)	12 (19.0)	
Other	10 (6.1)	4 (3.96)	4 (6.35)	
Unknown	1	1 (0.99)	0	
Disease group				
UCTD	43 (26.2)	34 (33.7)	9 (14.3)	< 0.0001
SLE	67 (40.9)	40 (39.6)	27 (42.9)	
MCTD	13 (7.93)	4 (3.96)	9 (14.3)	
SS	20 (12.2)	6 (5.94)	14 (22.2)	
IIM	8 (4.88)	6 (5.94)	2 (3.17)	
SSc	13 (7.93)	11 (10.9)	2 (3.17)	
Concomitant medication use				
Oral prednisolone	45 (27.3)	28 (27.7)	17 (27.0)	0.918
Anti-malarial*	96 (58.5)	64 (63.4)	32 (50.8)	0.112
Immunosuppressant**	47 (28.7)	28 (27.7)	19 (30.2)	0.737
Biological agent (within 6 months)	2 (1.22)	2 (1.98)	0	0.261
Daily dose prednisolone (mg)	7.5 (5.0, 10)	7.5 (5.0, 10)	7.5 (6.25, 10)	0.552
Autoantibodies				
dsDNA	46 (28.0)	24 (23.8)	22 (34.9)	0.122
Ro/SS-A	49 (29.9)	18 (17.8)	31 (49.2)	< 0.0001
La/SS-B	23 (14.0)	9 (8.91)	14 (22.2)	0.017
Smith	23 (14.0)	5 (4.95)	18 (28.6)	< 0.0001
RNP	39 (23.8)	13 (12.9)	26 (41.3)	< 0.0001
Chromatin	33 (20.1)	9 (8.91)	24 (38.1)	< 0.0001
Rheumatoid factor	37 (22.6)	12 (11.9)	25 (29.7)	< 0.0001
CCP	7 (4.3)	3 (2.97)	4 (6.35)	0.298
SSC-specific†	15 (9.1)	11 (10.9)	4 (6.35)	0.326
Jo-1	4 (2.4)	3 (2.97)	1 (1.59)	0.577
Clinical features				
Photosensitivity	64 (39.3)	43 (42.6)	21/62 (33.9)	0.174
Myositis-specific rash	10 (6.10)	7 (6.93)	3 (4.76)	0.419
Discoid lesion	6 (3.68)	3 (2.97)	3/62 (4.84)	0.414
Mucosal ulcers	63 (38.7)	41 (40.6)	22/62 (35.5)	0.315
Raynaud’s syndrome	87 (53.4)	55 (54.5)	32/62 (51.6)	0.424
Inflammatory arthritis	75 (46.0)	42/100 (42.0)	33 (52.4)	0.129
Renal disease‡	24 (14.9)	13/99 (13.1)	11/62 (17.7)	0.281
Neurological disease ‡	4 (2.44)	2 (1.98)	2 (3.17)	0.498
Haematological disorder‡	74 (46.3)	35/99 (35.4)	39/61 (63.9)	< 0.0001

Data are shown as the *n*(%) or median (IQR). ISG = interferon-stimulated gene

*Hydroxychloroquine, mepacrine or chloroquine phosphate

**Azathioprine, methotrexate, mycophenolate mofetil, ciclosporine, tacrolimus

†Anti-Scl70, anti-centromere and anti-RNA-polymerase III

‡As the per modified 1997 ACR Classification criteria for SLE

**Fig. 1:**
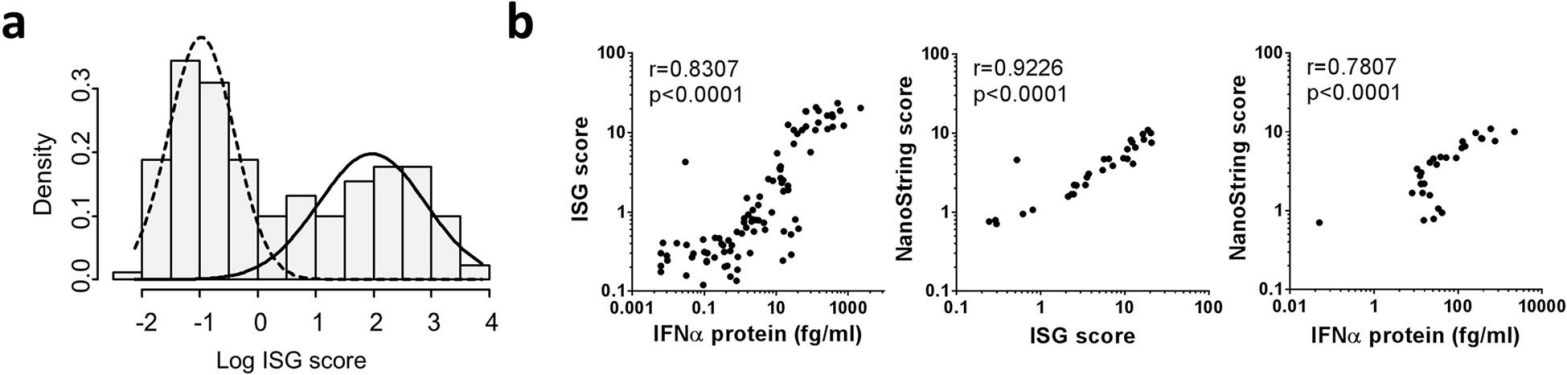
Distribution of the 6-gene ISG score in the SARD cohort. **a** The log-transformed 6-gene ISG score shows a bimodal distribution. **b** The 6-gene ISG score correlates both with measurement of IFN protein and with a more extensive 30-gene score measured using NanoString. Spearman’s *r* is shown

In the whole cohort the median (IQR) ISG score was 0.8115 (0.3155, 7.052). The log-transformed ISG score had a bimodal distribution (Fig. 1a). Using an ISG score cut-off of > 2.466 [11], 63 (38.4%) patients had a positive score. A finite mixture model was also used to group patients into IFN-high and IFN-low on the basis of the bimodal distribution and compared directly to our pre-validated cut-off. Using this approach, all IFN-low patients were classified as ISG score negative. Conversely, 9 patients were classified as IFN-high, but ISG score negative (see Additional file 1: Table S2).

In a subset of 92 patients, the IFNα plasma protein level was also measured using Simoa as previously described [14]. The median (IQR) IFNα protein concentration was 3.178 (0.361, 26.3) fg/ml. Using a cut-off of 10 fg/ml [9], 38/93 (40.9%) patients had a positive score. There was an excellent correlation between IFNα protein levels and the 6 gene RT-qPCR ISG score (0.8307, *p* < 0.0001). The 30 gene NanoString panel was measured in a subgroup of 30 patients, and there were excellent correlations between NanoString and the 6-gene RT-qPCR score (*r* = 0.9226, *p* < 0.0001) and between NanoString and IFNα protein (*r* = 0.7807, *p* < 0.0001) (Fig. 1b).

**Fig. 2:**
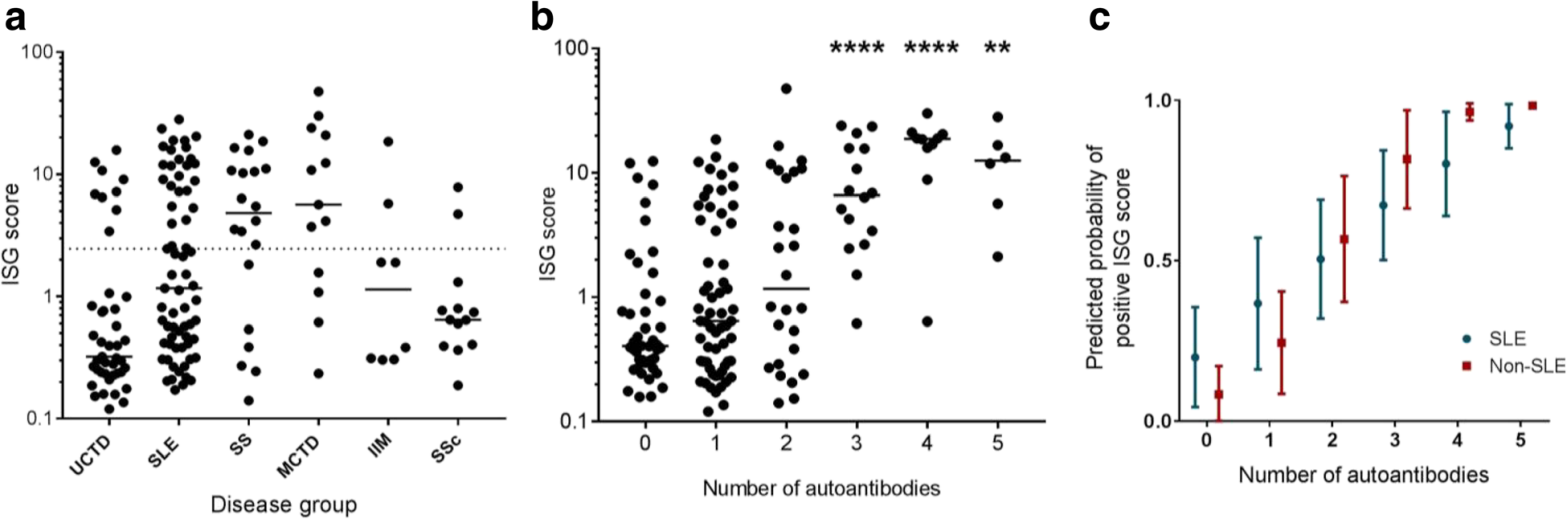
The ISG score is increased in a subset of SARD patients. **a** The number of patients with a positive ISG score varies between disease groups. Each data point represents a single subject according to their clinical diagnosis. The horizontal line shows the 95th centile for healthy subjects (ISG score of 2.466). **b** The ISG score is increased in patients with a greater number of autoantibodies. The total number of autoantibodies (excluding ANA) detected in each patient is shown. Comparisons are made against patients with specific autoantibodies (Dunn’s multiple comparison’s test), **p < 0.01, ****p < 0.0001. **c** The graph shows the predicted probabilities of a positive ISG score according to whether the patients have SLE (blue) or another SARD (red). These were obtained using logistic regression models adjusted for age, gender, ethnicity and concomitant mediation. The points and error bars show the mean and standard deviation of predicted probabilities

The number of patients with a positive ISG score varied between disease groups (Fisher’s exact test *p* < 0.001) (Fig. 2a and Table 1). Patients with SS and MCTD were most likely to have a positive ISG score (14/20, [70%] and 9/13 [69.2%], respectively) whilst positive ISG scores were less frequent in patients with SSc and UCTD (2/13 [15.4%] and 9/43 [20.9%], respectively). The distribution of ISG scores was similar when the relevant classification criteria were applied in lieu of the physician diagnosis (see Additional file 1: Figure S1).

In univariate logistic regression models, ISG score positivity was significantly associated with MCTD (OR [95% CI] 8.50 [2.12, 34.0]), SLE (2.55 [1.06, 6.16]) and SS (8.81 [2.64, 29.4]). In a multivariable model which also included medication (concomitant use (Y/N) of steroids, immunosuppressants and anti-malarials), these significant associations remained. We also noted an inverse association with age (0.97, [0.94, 0.998]), Caucasian ethnicity (0.38 [0.16, 0.91]) and anti-malarial use (0.41 [0.16, 1.04]).

### Higher ISG expression is associated with haematological abnormalities in SARDs

In the whole cohort, we found a higher frequency of haematological abnormalities, as defined by the 1997 Modified ACR Classification Criteria for SLE [15] in patients with a positive ISG score (39/61 [63.9]% vs. 35/99 [35.4]%, *p* < 0.0001). In addition, patients with a positive ISG score demonstrated lower haemoglobin concentration and lower total white cell count (WCC), lymphocyte and neutrophil counts (Table 2). The platelet count was also slightly lower in ISG score-positive patients, although this was not statistically significant. There was no association between ISG score positivity and other common SARD clinical features.

**Table 2:** Association between ISG score and haematological parameters

	ISG negative (*n* = 101)	ISG positive (*n* = 63)	*p* value
Haemolytic anaemia	1/99 (1.01)	1/60 (1.67)	0.614
Haemoglobin (mg/dl)	13.4 (12.5, 13.9)	12.9 (11.6, 13.3)	0.004
Total WCC (10^9^/l)	5.80 (4.55, 7.25)	4.95 (3.60, 5.80)	0.001
Lymphocyte count (10^9^/l)	1.62 (1.27, 2.10)	1.31 (0.96, 1.75)	0.002
Neutrophil count (10^9^/l)	3.39 (2.47, 4.51)	2.81 (1.99, 3.86)	0.020
Platelet count (10^9^/l)	253 (221, 311)	249 (191, 286)	0.078

Results are presented as the *n*(%) or median (IQR) as appropriate. Comparisons between groups were made using the Mann-Whitney U test (continuous variables) or Fisher’s exact test (categorical variables)

ISG = interferon-stimulated gene, WCC = white cell count

†As per the ACR SLE Classification criteria

In multivariable linear regression models which also included disease group, number of autoantibodies and medication use, the total WCC, lymphocyte count and neutrophil count all remained significantly inversely associated with the ISG score (see Additional file 1: Table S3).

### Autoantibodies against RNA proteins are associated with a positive ISG score

The association between the autoantibody profiles of patients was determined using logistic regression models. In unadjusted univariate models, the odds of being in the ISG score-positive group were significantly increased in patients with anti-Ro, anti-La, anti-Sm, anti-RNP and anti-chromatin antibodies and rheumatoid factor (Table 3). There were significant differences in the frequency of anti-Ro, anti-La, anti-Smith, anti-RNP, anti-chromatin, anti-dsDNA and SSc-specific antibodies between the clinical disease groups. In a fully-adjusted model (including disease group) anti-Smith, anti-chromatin and rheumatoid factor all remained significantly associated with the ISG score.

**Table 3:** Association between autoantibodies and ISG score using logistic regression models

	Odds ratio (95% CI)			
	Univariate model	Adjusted for age and gender	Adjusted model 1†	Adjusted model 2†
dsDNA	1.72 (0.862, 3.44)	1.45 (0.71, 2.97)	1.33 (0.576, 3.08)	1.47 (0.575, 3.74)
Ro (SS-A)	4.47 (2.20, 9.08)*	4.65 (2.24, 9.67)*	3.38 (1.37, 8.35)*	3.56 (1.32, 9.61)*
La (SS-B)	2.92 (1.18, 7.22)*	2.79 (1.11, 7.02)*	1.98 (0.602, 6.54)	2.50 (0.674, 9.26)
Smith	7.68 (2.68, 22.0)*	6.51 (2.23, 19.0)*	5.36 (1.61 17.8)*	8.35 (2.11, 32.7)*
RNP	4.76 (2.21, 10.2)*	4.07 (1.81, 9.14)*	4.11 (1.61, 10.5)*	4.08 (1.45, 11.5)*
Chromatin/nucleosome	6.29 (2.68, 14.8)*	5.57 (2.24, 13.9)*	5.13 (1.84, 14.3)*	8.09 (2.31, 28.4)*
Rheumatoid factor	4.88 (2.22, 10.7)*	5.53 (2.42, 12.6)*	7.77 (2.76, 21.9)*	7.26 (2.32, 22.7)*
Anti-CCP	2.31 (0.597, 8.91)	2.25 (0.577, 8.77)	1.46 (0.263, 8.07)	1.87 (0.272, 12.6)
SSc-specific ‡	0.835 (0.582, 1.20)	0.862 (0.592, 1.25)	1.00 (0.614, 1.64)	1.98 (0.237, 16.5)

*p < 0.05

ISG = interferon-stimulated gene

†Model 1: adjusted for age, gender, ethnicity and clinical diagnosis

Model 2: adjusted for above plus disease duration, anti-malarial, prednisolone and immunosuppressant use

‡Anti-Scl70, anti-centromere and anti-RNA-polymerase III

In patients with no specific autoantibodies (beyond ANA), the median (IQR) ISG score was 0.407 (0.303, 0.998) and there was a significant increase in ISG score as the total number of autoantibodies in each patient (defined as anti-dsDNA, anti-Smith, anti-RNP, anti-Ro, anti-La, anti-CCP, anti-chromatin/nucleosome or SSc-specific antibodies [anti-centromere, anti-Scl-70 or RNA polymerase III]) increased (Kruskal-Wallis, *p* = 0.0001) (Fig. 2b). In a multivariable model including disease group and medication use, each additional autoantibody increased the odd of having a positive ISG score by over 2-fold (unadjusted OR 2.44 [176, 3.36], adjusted OR 2.14 [1.49, 30.5]). The association between autoantibody number and ISG score was also determined for SLE patients alone compared to the rest of the SARD cohort. In both cases, a significant association was observed (OR 1.87 [1.18, 2.96] and OR 4.62 [2.35, 90.6] respectively, Fig. 2c).

ISG score-positive patients also had a higher median (IQR) ESR (erythrocyte sedimentation rate) (14.0 [8, 35.5] vs. 8.0 [5, 17.5] mm/h, *p* = 0.007) but not CRP (serum C-reactive protein) concentration (2.0 [1.0, 4.0] vs. 1.5 [1.0, 5.0] mg/dl, *p* = 0.7196). There was no association between a positive ISG score and history of low C3 or low C4 complement.

### Whole blood RNA-Seq analysis

**Fig. 3:**
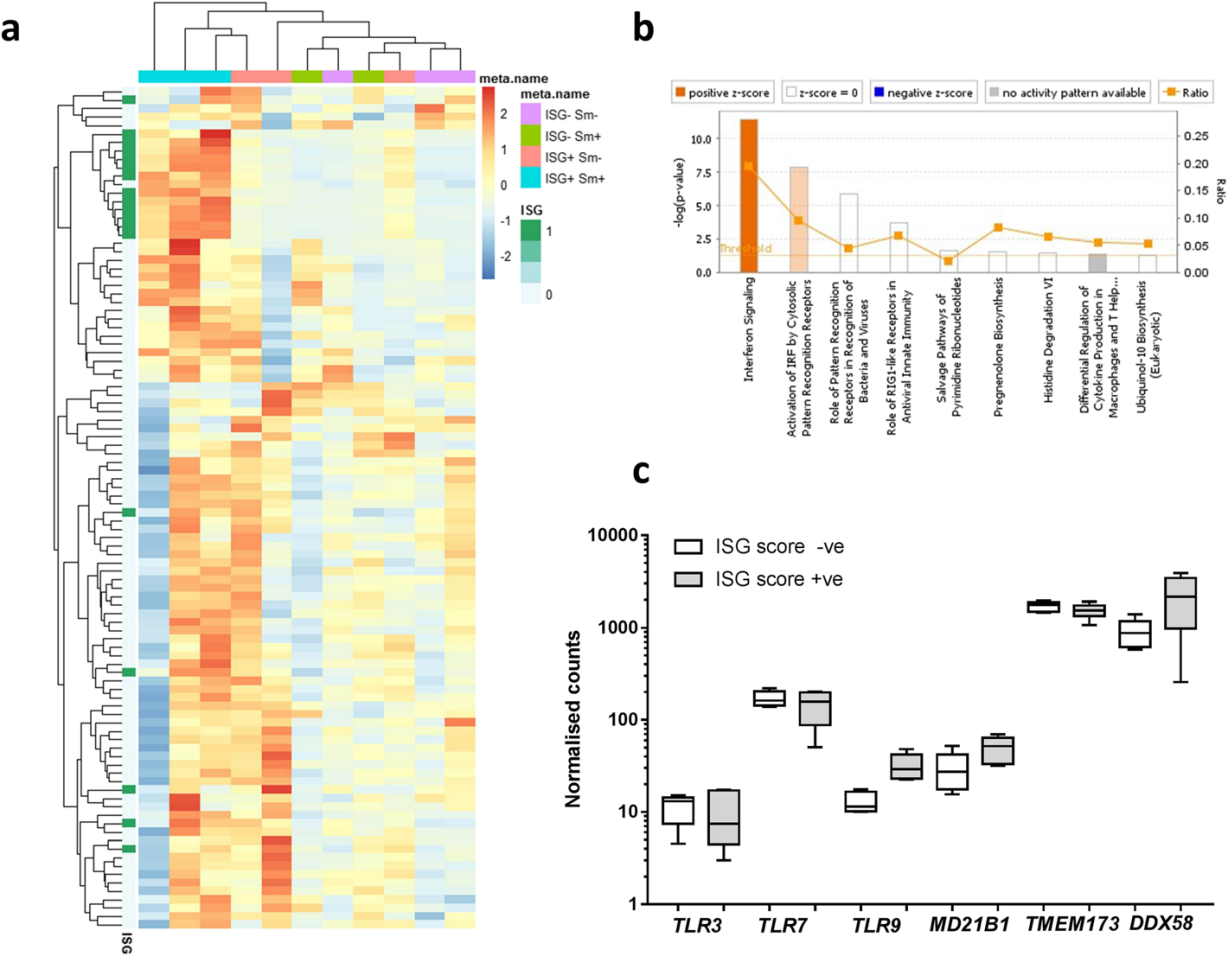
Whole blood transcriptome analysis of ISG-positive and ISG-negative SLE patients. **a** Heatmap of the 100 genes with the greatest variance. Upregulated genes are shown in red; downregulated genes are shown in blue. The patient samples are hierarchically clustered (Euclidean distance) over all coding genes. The heatmap is annotated to show known ISGs (dark green). Each patient sample is annotated according to the ISG score (positive or negative) and the anti-Sm antibody status (positive or negative). **b** Gene ontology analysis showing the canonical pathways that are over-represented in the ISG score-positive patients compared to the negative patients. **c** The expression of nucleic acid receptors (NARs) within the RNA-Seq dataset between the ISG score-positive (*n* = 6) and ISG score-negative (*n* = 5) patients

An exploratory RNA-Seq analysis was performed on samples from 12 SLE participants from 4 ‘cohorts’: 3 ISG positive/anti-Sm positive; 3 ISG positive/anti-Sm negative; 3 ISG-negative/anti-Sm-positive participants and 3 ISG-negative/anti-Sm-negative. One ISG-negative/anti-Sm-positive patient failed QC and was removed from the analysis. Anti-Sm was selected as it was most strongly related to the ISG score, allowing analysis of patients who were both ISG and Sm positive (‘double positive’), and patients with anti-Sm without a positive ISG. Hierarchical clustering of all coding transcripts was conducted (after removal of any globin/HLA genes). This demonstrated some similarities between patients belonging to the groupings described (Fig. 3a). Strikingly, of the 100 genes showing the greatest variance across all 11 samples, only a small number were ISGs. In addition, although the 3 ISG positive/anti-Sm positive patients shared high expression of the ISGs, 1 of the 3 patients (lane 1) showed markedly different expression levels of the other genes.

Gene ontology analysis of over-represented canonical biological pathways, between the ISG positive (*n* = 6) and negative (*n* = 5) participants, identified differential expression of nucleic acid receptors (NARs). The GO terms with the greatest enrichment were (i) interferon signalling, (ii) activation of IRF by cytosolic pattern recognition receptors and (iii) role of pattern recognition receptors in recognition of bacteria and viruses (Fig. 3b). The expression of a number of NARs was increased in ISG score-positive patients, and a group of these were then taken forward for further study (Fig. 3c).

### Expression of nucleic acid receptors (NARs) differs between disease groups and is associated with the ISG score

**Fig. 4:**
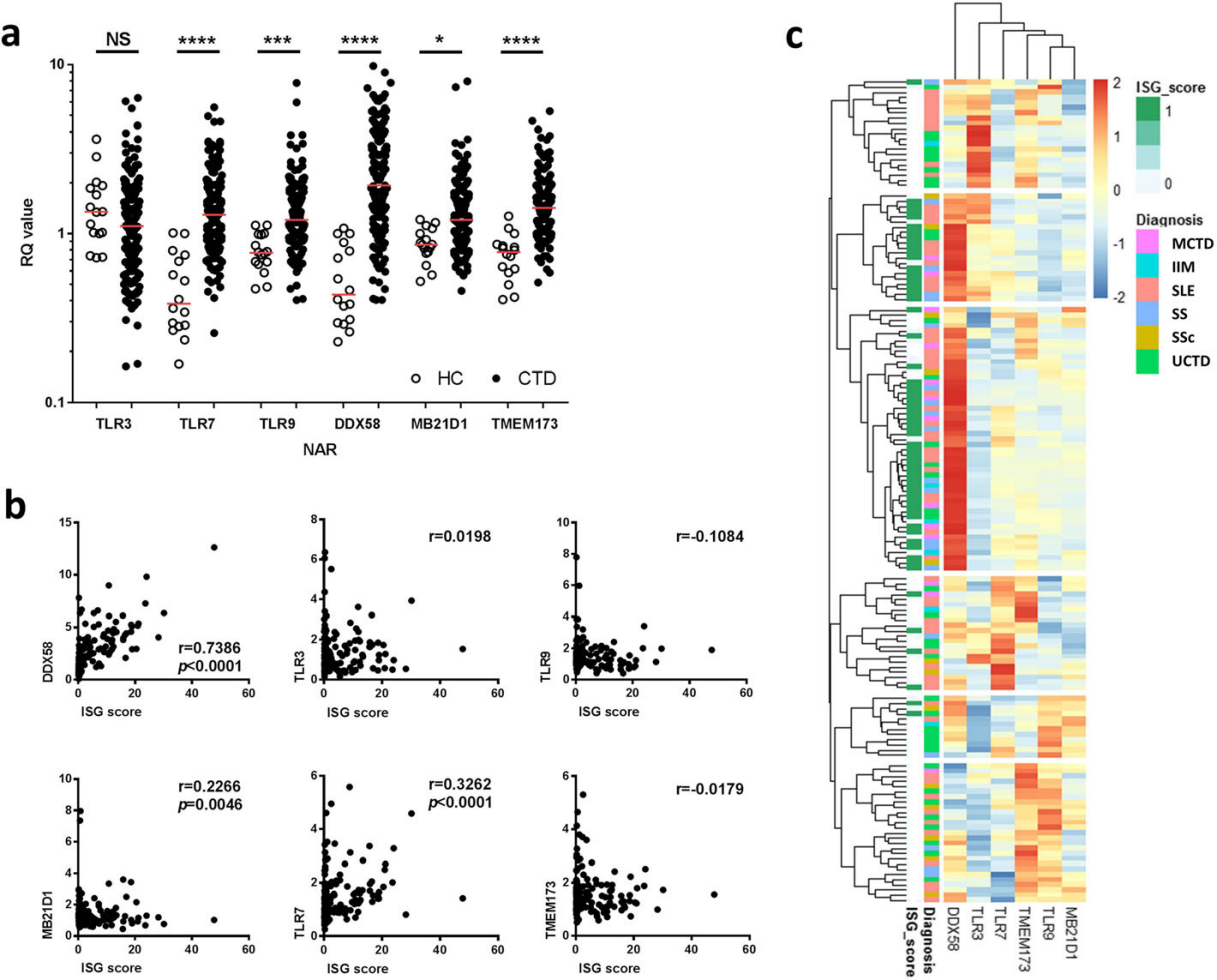
Nucleic acid receptors are differentially expressed in patients with SARDs. **a** CTD patients have increased expression of all of the NARs except TLR3. Comparisons were made using Dunn’s multiple comparison’s test. *p < 0.05, ***p < 0.001, ****p < 0.0001. RQ = relative quantification of gene expression. **b** Heatmap showing the relative expression of each of the 6 NARs. Each row represents a single patient and is K-means clustered into 6 groups. The rows are annotated by diagnostic group and by ISG score (ISG score-positive patients in green). **c** Correlation between the NAR expression and ISG score in the combined CTD cohort. The graphs show the Spearman *r* for each NAR

The expression of 6 NARs (TLR3, TLR7, TLR9, DDX58 [RIG-I], MB21D1 [c-GAS] and TMEM173 [STING]) was measured in 155 patients and 16 HC (healthy controls were 14/16 [87.5]% female with a median age of 42 years). Expression of all NARs (with the exception of TLR3) was increased in SARDs compared to HC (Fig. 4a). There was a strong correlation between the ISG score and DDX58 expression (*r* = 0.7386, *p* < 0.0001) and modest correlations between the ISG score and TLR7 and MB21D1 (*r* = 0.3262, *p* < 0.0001 and *r* = 0.2266, *p* = 0.005, respectively) (Fig. 4b). Using logistic regression DDX58 and TLR7 were associated with a positive ISG score (OR 3.23 [2.25, 4.67] and 1.68 [1.14, 2.46] with DDX58 remaining significant in a multivariable model containing disease group (OR 3.69, [2.32, 5.85])).

Expression of all of the NARs (except TLR3) was higher in patients compared to healthy controls according to disease phenotype (see Additional file 1: Figure S2). In linear regression models of log-adjusted NAR expression, patients with anti-Ro, anti-RNP, anti-Smith and anti-chromatin antibodies had significantly increased DDX58 expression (see Additional file 1: Table S4). These associations remained significant after adjustment for age and gender, but were no longer significant when the ISG score was included in the model (data not shown). Patients with anti-Ro antibodies also had significantly lower expression of TLR9 and MB21D1 which remained significant in a multivariable model that included ISG score (β − 0.173 [− 0.233, − 0.015] and − 0.190 [− 0.346, − 0.034].

In an exploratory analysis, we identified clusters of patients based on the expression of the 6 NARs (Fig. 4c). Of those patients with a positive ISG score and increased DDX58 expression, a subgroup also had increased expression of TLR3. In contrast, patients with increased TMEM173 also had increased expression of either TLR7 or TLR9, but not both.

### TLR3, TLR9 and MB21D1 (c-GAS) expression are independently associated with neutrophil counts

**Fig. 5:**
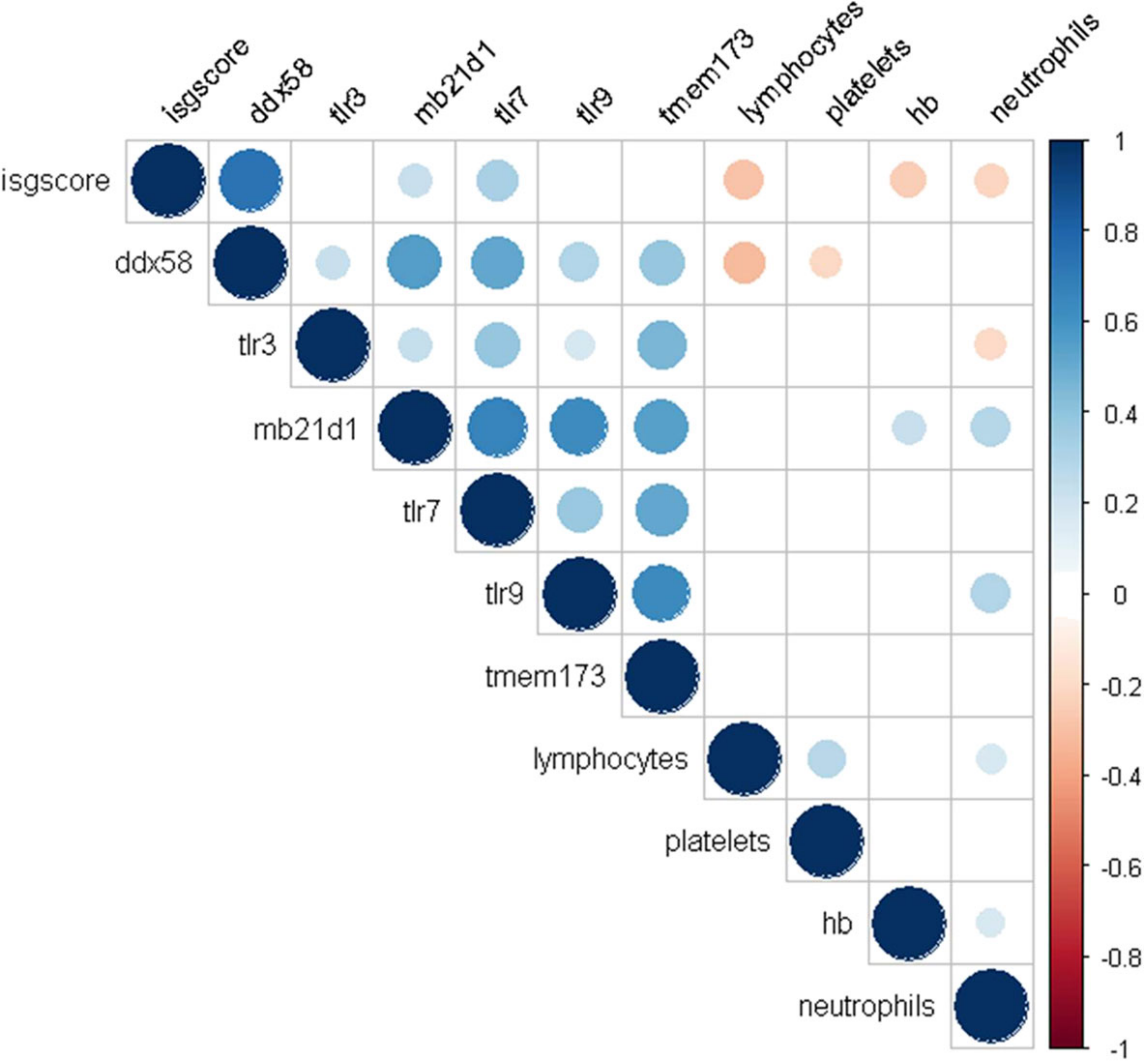
Correlation between the ISG score, NARs and haematological parameters. The figure shows a Spearman correlation matrix for the ISG score, NAR expression and haematological parameters. Only statistically significant (*p* < 0.05) parameters are shown. The size of the dot represents the strength of correlation (Spearman’s *r*); blue = positive correlation, red = negative correlation. Hb = haemoglobin

Significant correlations between a number of the NARs and haematological parameters (haemoglobin, lymphocyte count, neutrophil count and platelets) were identified (Fig. 5). Interestingly, both positive and negative correlations were observed for neutrophils. Linear regression models identified that TLR3 was negatively associated, whilst TLR9 and MB21D1 were positively associated, with neutrophil counts even after adjusted for prednisolone use and the ISG score (Table 4).

**Table 4:** Association between NAR expression and neutrophil count

	Beta coefficient (95% CI)			
	Univariate model	Adjusted model 1†	Adjusted model 2†	Adjusted model 3†
TLR3	−0.113 (−0.206, −0.019)*	−0.121 (−0.212, −0.030)*	− 0.116 (− 0.203, − 0.29)*	−0.107 (− 0.194, − 0.022)*
TLR9	0.161 (0.072, 0.250)*	0.138 (0.048, 0.229)*	0.102 (0.011, 0.193)*	0.095 (0.006, 0.184)*
MB21D1	0.151 (0.065, 0.237)*	0.126 (0.039, 0.213)*	0.096 (0.008, 0.184)*	0.100 (0.015, 0.186)*

Linear regression models of log-normalised neutrophil counts

†Model 1: adjusted for age, gender, ethnicity (Caucasian vs non-Caucasian) and clinical diagnosis

Model 2: as above plus adjusted for prednisolone, anti-malarial and immunosuppressant use

Model 3: as above plus adjustment for ISG score

*p < 0.05

## Discussion

In this mixed SARD cohort, we used multiple methodologies to identify subgroups of patients with shared immunopathological phenotypes despite different clinical disease manifestations. Interestingly, in our multivariable regression models, type I IFN pathway activity was more strongly associated with specific autoantibodies and haematological parameters than clinical disease subtypes or individual clinical features.

Since type I interferon was first reported to be present in the serum of SLE patients in 1975 [16], a number of studies have examined type I IFN expression in patients with SARDs [17]. A panel of downstream ISGs, termed the ‘interferon signature’, is commonly used as a surrogate of IFNα expression. In this study, we used our established 6-probe RT-qPCR ISG assay [11] which has been extensively validated in patients with monogenic interferonopathies [18]. Although our patient and healthy control groups varied in terms of age, we have previously reported that our ISG score is not associated with age [11]. We also employed NanoString technology assessing 30 probes of interest. The significant correlation between these measures demonstrates that either method may be used to assess ISGs, with NanoString potentially offering a less operator-dependent technique, and thus greater potential for widespread clinical use. We also demonstrated a strong correlation with IFNα protein using a high-sensitivity digital ELISA, which could detect IFNα protein in the fg/ml range. This gives confidence that our six-gene panel is measuring the downstream effects of in vivo IFNα activity. In 6 (6.5%) patients, we observed a difference between the ISG score and digital ELISA and these patients are the subject of further study. Similarly to El-Sherbiny et al. [19], we also observed that the ISG score has a clear bimodal distribution. Although we utilised our pre-defined threshold for the ISG score, this approach and the results of the finite mixture model were highly concordant.

In our cohort, increased IFN expression was identified in 38% of patients, with a subpopulation in each clinical disease group demonstrating elevated levels. The overall number of ISG score-positive patients is lower than that observed in other autoimmune populations, perhaps reflecting the fact that we report an established mixed SARD cohort (including patients with UCTD), or that the patients had clinically stable disease, and that our cohort was predominantly Caucasian. We confirmed the findings of others that ISG score-positive patients were younger, but that there was no association with disease duration [10]. In a large study by Weckerle et al., IFNα activity was significantly increased in patients of non-European ancestry [20]. The relationship between IFNα and disease activity is less clear; in patients with SLE, some groups have reported an association [21, 22] whilst others have not [23, 24]. Most recently, a study of the whole blood ISG signature in SLE patients showed remarkable stability over time with no association with changes in disease activity [25]. This suggests that the IFN signature may be used to identify a phenotypic subpopulation of SARD patients in cross-sectional studies.

It has been proposed that increased interferon expression may pre-date the development of SARDs. In a study by Wither et al., increased IFN expression was identified in asymptomatic patients with a positive ANA [26]. Furthermore, a positive IFN score may predict those patients who go on to develop SLE or SS. In a study of 118 patients considered to be at risk for developing a SARDs, the IFN score was greater in those who progressed to a SARD than those who did not [27]. We also identified an ISG-positive subgroup in our UCTD patients with established disease.

In our mixed SARD cohort, we observed that this type I IFN signature was associated with haematological indices, but not with other autoimmune features. An inverse association between lymphocyte count and IFN score has been reported previously [19], but in our cohort, we also found associations with neutrophils, haemoglobin and a trend towards a lower platelet count. In the general population, viral infections with a corresponding IFN response are associated with lymphopenia [28]. Similarly, chronic IFNα exposure results in reduced haemoglobin, neutrophils and lymphocytes in patients with chronic hepatitis C, supporting a direct role for IFNα in the haematological abnormalities observed in our cohort [29].

The proportion of patients with haemolytic anaemia was very small, and we only observed a trend towards lower platelet counts; this is in contrast to patients with monogenic type I interferonopathies in whom thrombocytopenia is often the most common haematological manifestation [30, 31]. In addition, iatrogenic thrombocytopenia has been reported following interferon-β therapy [32]. The mechanism by which IFN may result in these haematological abnormalities is yet to be fully elucidated; however, it is likely that a number of mechanisms are involved including increased lymphocyte migration to tissue and reduced egress from lymph nodes [33].

In this larger clinical cohort, we corroborated our previous findings of a positive association between elevated type I interferon and the RNA-associated autoantibodies anti-Ro, anti-La, anti-Sm and anti-RNP [9]. Additionally, we observed an association between anti-chromatin/nucleosome antibodies and rheumatoid factor and a positive ISG score which appeared to be independent of the clinical diagnosis. Importantly, anti-chromatin antibodies were associated with increased IFNα whilst anti-dsDNA antibodies were not, suggesting that the DNA-histone complex may be more interferonogenic than DNA alone. This observation is in contrast with the study by Kirou et al. which found an association between IFNα and anti-dsDNA titres in SLE patients, although this may have been confounded by increased disease activity [21]. RNA-containing immune complexes appear to directly increase IFNα production in plasmacytoid dendritic cells (pDCs) in a mechanism that is dependent on both toll-like receptors (TLRs) and the Fc gamma receptor IIa [34], but may also trigger IFNα indirectly via effects on neutrophils [35].

We observed increased expression of all of the nuclear receptors, except TLR3, in whole blood of patients with SARDs compared to healthy controls. This supports the study by Chauhan et al. which identified increased PBMC TLR7 and TLR9 expression in SLE patients compared to healthy controls [36]. However, in our cohort, DDX58/RIG-I, MB21D1 and TLR7 expression were associated with the ISG score which may reflect our measurements in whole blood which does not allow adjustment for immune cell heterogeneity. Maria et al. reported that TLR7 was increased and TLR9 decreased in the pDCs and monocytes of IFN-positive SS patients [37]. In addition, DDX58/RIG-I was also increased in monocytes from IFN-positive patients. Similarly in childhood SLE, increased expression of TLR7 and a number of RIG-like receptors (including DDX58) has been reported in IFN score-positive patients [38].

Unsupervised hierarchical clustering of NAR expression identified groups of ISG-score-negative patients with high TLR3 expression, or high TLR9 and/or TMEM173/STING expression. The potential roles of these receptors in IFN-low subgroups warrant further investigation. Interestingly, although TLR3 was not increased in SARD patients compared to controls, there was a significant independent negative association with neutrophil count (in opposition to MB21D1 and TLR9) suggesting that non-IFN mechanisms might be involved. In contrast, TLR3 ligation in mice has been demonstrated to result in increased neutrophil numbers suggesting that increased TLR expression and increased TLR3 signalling may not explain this association [39].

## Conclusions

In summary, transcriptomic and protein studies demonstrate that for subpopulations of the SARDs there are overlapping IFN-related biomarkers which may represent shared molecular drivers of disease and are more associated with specific disease features and ENA than clinical diagnoses. We also noted that NAR expression differs between ISG-positive and ISG-negative patients which may point to different immunopathological mechanisms between ISG “immunophenotypes”. Our observations suggest that the molecular taxonomy of a mixed SARD cohort is inconsistent with the clinical phenotype. A targeted approach to treatment of patients with a shared immunopathogenesis may therefore offer advantages over the current classification of disease.

## Additional file

Additional file 1: Supplementary methods (DOCX 1004 kb)

## References

[1] Banchereau R, Hong S, Cantarel B, Baldwin N, Baisch J, Edens M (2016). Personalized immunomonitoring uncovers molecular networks that stratify lupus patients. Cell.

[2] Barturen G, Beretta L, Cervera R, Van VR, Alarcon-Riquelme ME (2018). Moving towards a molecular taxonomy of autoimmune rheumatic diseases. Nat Rev Rheumatol.

[3] Ronnblom L, Alm GV, Eloranta ML (2011). The type I interferon system in the development of lupus. Semin Immunol.

[4] Higgs BW, Liu Z, White B, Zhu W, White WI, Morehouse C (2011). Patients with systemic lupus erythematosus, myositis, rheumatoid arthritis and scleroderma share activation of a common type I interferon pathway. Ann Rheum Dis.

[5] Brkic Z, Maria NI, van Helden-Meeuwsen CG, van de Merwe JP, van Daele PL, Dalm VA (2013). Prevalence of interferon type I signature in CD14 monocytes of patients with Sjogren's syndrome and association with disease activity and BAFF gene expression. Ann Rheum Dis.

[6] Manry J, Laval G, Patin E, Fornarino S, Itan Y, Fumagalli M (2011). Evolutionary genetic dissection of human interferons. J Exp Med.

[7] Rice GI, Del Toro DY, Jenkinson EM, Forte GM, Anderson BH, Ariaudo G (2014). Gain-of-function mutations in IFIH1 cause a spectrum of human disease phenotypes associated with upregulated type I interferon signaling. Nat Genet.

[8] Hua J, Kirou K, Lee C, Crow MK (2006). Functional assay of type I interferon in systemic lupus erythematosus plasma and association with anti-RNA binding protein autoantibodies. Arthritis Rheum.

[9] Rodero MP, Decalf J, Bondet V, Hunt D, Rice GI, Werneke S (2017). Detection of interferon alpha protein reveals differential levels and cellular sources in disease. J Exp Med.

[10] Mathian Alexis, Mouries‐Martin Suzanne, Dorgham Karim, Devilliers Hervé, Barnabei Laura, Ben Salah Elyès, Cohen‐Aubart Fleur, Garrido Castillo Laura, Haroche Julien, Hie Miguel, Pineton de Chambrun Marc, Miyara Makoto, Sterlin Delphine, Pha Micheline, Lê Thi Huong Du, Rieux‐Laucat Frédéric, Rozenberg Flore, Gorochov Guy, Amoura Zahir (2019). Monitoring Disease Activity in Systemic Lupus Erythematosus With Single‐Molecule Array Digital Enzyme‐Linked Immunosorbent Assay Quantification of Serum Interferon‐α. Arthritis & Rheumatology.

[11] Rice GI, Forte GM, Szynkiewicz M, Chase DS, Aeby A, Abdel-Hamid MS (2013). Assessment of interferon-related biomarkers in Aicardi-Goutieres syndrome associated with mutations in TREX1, RNASEH2A, RNASEH2B, RNASEH2C, SAMHD1, and ADAR: a case-control study. Lancet Neurol.

[12] Meyer S, Woodward M, Hertel C, Vlaicu P, Haque Y, Karner J (2016). AIRE-deficient patients harbor unique high-affinity disease-ameliorating autoantibodies. Cell.

[13] Rusinova I, Forster S, Yu S, Kannan A, Masse M, Cumming H (2013). Interferome v2.0: an updated database of annotated interferon-regulated genes. Nucleic Acids Res.

[14] Llibre A, Bondet V, Rodero MP, Hunt D, Crow YJ, Duffy D. Development and validation of an ultrasensitive single molecule array digital enzyme-linked immunosorbent assay for human interferon-alpha. J Vis Exp. 2018;136. 10.3791/57421. 10.3791/57421 PMC6101729 29985347

[15] Hochberg MC (1997). Updating the American college of rheumatology revised criteria for the classification of systemic lupus erythematosus. Arthritis Rheum.

[16] Skurkovich SV, Eremkina EI (1975). The probable role of interferon in allergy. Ann Allergy.

[17] Ronnblom L, Eloranta ML (2013). The interferon signature in autoimmune diseases. Curr Opin Rheumatol.

[18] Rice GI, Melki I, Fremond ML, Briggs TA, Rodero MP, Kitabayashi N (2017). Assessment of type I interferon signaling in pediatric inflammatory disease. J Clin Immunol.

[19] El-Sherbiny YM, Psarras A, Yusof MYM, Hensor EMA, Tooze R, Doody G (2018). A novel two-score system for interferon status segregates autoimmune diseases and correlates with clinical features. Sci Rep.

[20] Weckerle CE, Franek BS, Kelly JA, Kumabe M, Mikolaitis RA, Green SL (2011). Network analysis of associations between serum interferon-alpha activity, autoantibodies, and clinical features in systemic lupus erythematosus. Arthritis Rheum.

[21] Kirou KA, Lee C, George S, Louca K, Peterson MG, Crow MK (2005). Activation of the interferon-alpha pathway identifies a subgroup of systemic lupus erythematosus patients with distinct serologic features and active disease. Arthritis Rheum.

[22] Rose T, Grutzkau A, Hirseland H, Huscher D, Dahnrich C, Dzionek A (2013). IFNalpha and its response proteins, IP-10 and SIGLEC-1, are biomarkers of disease activity in systemic lupus erythematosus. Ann Rheum Dis.

[23] Kennedy WP, Maciuca R, Wolslegel K, Tew W, Abbas AR, Chaivorapol C (2015). Association of the interferon signature metric with serological disease manifestations but not global activity scores in multiple cohorts of patients with SLE. Lupus Sci Med.

[24] Landolt-Marticorena C, Bonventi G, Lubovich A, Ferguson C, Unnithan T, Su J (2009). Lack of association between the interferon-alpha signature and longitudinal changes in disease activity in systemic lupus erythematosus. Ann Rheum Dis.

[25] Petri M, Fu W, Ranger A, Allaire N, Cullen P, Magder LS (2019). Association between changes in gene signatures expression and disease activity among patients with systemic lupus erythematosus. BMC Med Genet.

[26] Wither J, Johnson SR, Liu T, Noamani B, Bonilla D, Lisnevskaia L (2017). Presence of an interferon signature in individuals who are anti-nuclear antibody positive lacking a systemic autoimmune rheumatic disease diagnosis. Arthritis Res Ther.

[27] Md Yusof MY, Psarras A, El-Sherbiny YM, Hensor EMA, Dutton K, Ul-Hassan S (2018). Prediction of autoimmune connective tissue disease in an at-risk cohort: prognostic value of a novel two-score system for interferon status. Ann Rheum Dis.

[28] Schattner A, Meshorer A, Wallach D (1983). Involvement of interferon in virus-induced lymphopenia. Cell Immunol.

[29] Schmid M, Kreil A, Jessner W, Homoncik M, Datz C, Gangl A (2005). Suppression of haematopoiesis during therapy of chronic hepatitis C with different interferon alpha mono and combination therapy regimens. Gut.

[30] Crow YJ, Manel N (2015). Aicardi-Goutieres syndrome and the type I interferonopathies. Nat Rev Immunol.

[31] Briggs TA, Rice GI, Adib N, Ades L, Barete S, Baskar K (2016). Spondyloenchondrodysplasia due to mutations in ACP5: a comprehensive survey. J Clin Immunol.

[32] Koudriavtseva T, Plantone D, Renna R, Mandoj C, Giannarelli D, Mainero C (2015). Interferon-beta therapy and risk of thrombocytopenia in multiple sclerosis patients. Neurol Sci.

[33] Kamphuis E, Junt T, Waibler Z, Forster R, Kalinke U (2006). Type I interferons directly regulate lymphocyte recirculation and cause transient blood lymphopenia. Blood.

[34] Lovgren T, Eloranta ML, Kastner B, Wahren-Herlenius M, Alm GV, Ronnblom L (2006). Induction of interferon-alpha by immune complexes or liposomes containing systemic lupus erythematosus autoantigen- and Sjogren's syndrome autoantigen-associated RNA. Arthritis Rheum.

[35] Lande R, Ganguly D, Facchinetti V, Frasca L, Conrad C, Gregorio J (2011). Neutrophils activate plasmacytoid dendritic cells by releasing self-DNA-peptide complexes in systemic lupus erythematosus. Sci Transl Med.

[36] Chauhan SK, Singh VV, Rai R, Rai M, Rai G (2013). Distinct autoantibody profiles in systemic lupus erythematosus patients are selectively associated with TLR7 and TLR9 upregulation. J Clin Immunol.

[37] Maria NI, Steenwijk EC, IJpma AS, van Helden-Meeuwsen CG, Vogelsang P, Beumer W (2017). Contrasting expression pattern of RNA-sensing receptors TLR7, RIG-I and MDA5 in interferon-positive and interferon-negative patients with primary Sjogren's syndrome. Ann Rheum Dis.

[38] Wahadat MJ, Bodewes ILA, Maria NI, van Helden-Meeuwsen CG, van Dijk-Hummelman A, Steenwijk EC (2018). Type I IFN signature in childhood-onset systemic lupus erythematosus: a conspiracy of DNA- and RNA-sensing receptors?. Arthritis Res Ther.

[39] Downes JE, Marshall-Clarke S (2010). Innate immune stimuli modulate bone marrow-derived dendritic cell production in vitro by toll-like receptor-dependent and -independent mechanisms. Immunology.

