# Supplementary material for: Type I interferon in patients with systemic autoimmune rheumatic disease is associated with haematological abnormalities and specific autoantibody profiles
Source: Arthritis Res Ther. 2019 Jun 14;21:147. doi: 10.1186/s13075-019-1929-4 (PMC6567906; doi:10.1186/s13075-019-1929-4)
Supplement: Supplementary file 1 — Supplementary methods (DOCX 1004 kb) [file 13075_2019_1929_MOESM1_ESM.docx]

Supplementary Methods

Gene expression analysis by RNASeq

RNA was extracted as described above and RNA integrity was analysed using the Agilent 2100 Bioanalyzer. mRNA purification and fragmentation, complementary DNA synthesis and target amplification were performed using the Illumina® TruSeq RNA Sample Preparation Kit (Illumina). Pooled cDNA libraries were sequenced using HiSeq 2000 Illumina® platform (Illumina). The RNA-Seq workflow was as follows: quality assessment by FastQC (http://www.bioinformatics.babraham.ac.uk/projects/fastqc/), quality filtering with Trimmomatic (PMID: 24695404), read mapping to hg38 and counting into genes with STAR (PMID: 23104886) using annotation from GENCODE v24 (http://www.gencodegenes.org/), normalization and differential expression analysis with DESeq2 (PMID: 25516281). The statistical significance level adopted was q<0.05.

NanoString ISG analysis

Analysis of 30 genes and 3 housekeeping genes was conducted using the NanoString customer designed CodeSets according to the manufacturer’s recommendations (NanoString Technologies, Seattle, WA). 100ng of total RNA was loaded for each sample. Agilent Tapestation was used to assess the quality of the RNA. Data were processed with nSolver software (NanoString Technologies Seattle, WA)

Table S1: Probes used in NanoString analysis.

| Probes of interest | Reference probes |
| --- | --- |
| \| IFI27 \| \| --- \| \| IFI44L \| \| OTOF \| \| SIGLEC1 \| \| RSAD2 \| \| IFI44 \| \| IFIT1 \| \| ISG15 \| \| CMPK2 \| \| MX1 \| \| LY6E \| \| EPSTI1 \| \| SPATS2L \| \| IFI6 \| \| OAS1 \| \| HERC5 \| \| NRIR \| \| HES4 \| \| FBXO39 \| \| IRF7 \| \| DDX60 \| \| LAMP3 \| \| OASL \| \| IFIT5 \| \| LGALS3BP \| \| EIF2AK2 \| \| CXCL10 \| \| TRIM22 \| \| IFIH1 \| \| MT2A \| | HPRT1  NRDC OTUD5 |

Supplementary Data:

Table S1: Clinical characteristics of the cohort

The table describes the characteristics of the cohort, split by disease type. The groups were compared using the K-Wallis test and the P value is reported.

|  | MCTD  (n=13) | IIM  (n=8) | SLE  (n=67) | SS  (n=20) | SSc  (n=13) | UCTD  (n=43) | P value |
| --- | --- | --- | --- | --- | --- | --- | --- |
| Age (years) | 48.7 (40.2, 55.4) | 53.5 (50.9, 58.6) | 45.4 (50.9, 58.6) | 53.0 (40.1, 57.6) | 60.5 (59.4, 65.0) | 44.8 (33.6, 52.3) | 0.0002 |
| Gender (female) | 9 (69.2) | 8 (100) | 65 (97.1) | 19 (95.0) | 13  (100) | 41  (95.4) | 0.003 |
| Disease duration (years) | 7.13 (4.92, 20.3) | 3.67 (0.756, 6.49) | 11.3 (5.88, 16.9) | 6.54 (2.37, 15.7) | 7.29 (3.21, 15.9) | 3.65 (1.71, 7.14) | 0.0001 |
| Caucasian ethnicity | 10 (76.9) | 4 (50.0) | 51 (76.1) | 18 (90.0) | 11 (84.6) | 28 (65.1) | 0.164 |
| Anti-malarial | 8 (61.5) | 4 (50.0) | 50 (74.6) | 8 (40.0) | 2 (15.4) | 24 (55.8) | 0.001 |
| Immunosuppressant | 5 (38.5) | 3 (37.5) | 25 (37.3) | 4 (20.0) | 3 (23.1) | 7 (16.3) | 0.184 |
| Prednisolone | 6 (46.2) | 2 (25.0) | 26 (38.8) | 3 (15.0) | 3 (23.1) | 5 (11.6) | 0.017 |

Table S2: Classification of patients by ISG score as a binary outcome compared to a finite mixture model (FMM).

The patients were classified as ISG score positive or negative using a binary cut-off point based on the upper 95^th^ percentile of healthy controls. In the finite mixture model, patients were classified as IFN high or low on the basis of >0.9 probability. The FMM classified slightly more patients as being ISG score high than using the binary cut off.

|  |  | ISG score (binary) | |
| --- | --- | --- | --- |
|  |  | Negative | Positive |
| IFN status using FMM | Low | 92 | 0 |
|  | High | 9 | 63 |

Table S3: Association between ISG score and clinical features of CTD

We produced univariate and logistic regression models to determine the association between the haematological parameters and the ISG score. The dependent variable was log-transformed for total WCC, lymphocyte count and neutrophil count. After accounting for important confounders there was a modest but statistically significant inverse association between WCC, lymphocyte count and neutrophil count and the ISG score.

|  | Unadjusted | Age and gender adjusted | Age, gender and clinical diagnosis | Fully adjusted† |
| --- | --- | --- | --- | --- |
| Haemoglobin | -0.601 (-1.036, -0.166)* | -0.033 (-0.0612, -0.005)* | -0.0355 (-0.0667, -0.004)* | -0.018 (-0.052, 0.016) |
| Total WCC‡ | -0.014 (-0.022, -0.006)* | -0.016 (-0.024, -0.008)* | -0.018 (-0.027, -0.009)* | -0.014 (-0.02, -0.005)* |
| Lymphocyte count‡ | -0.018 (-0.027, -0.008)* | -0.017 (-0.027, -0.007)* | -0.016 (-0.027, -0.005)* | -0.017 (-0.029 , -0.004)* |
| Neutrophils count‡ | -0.012 (-0.022, -0.001)* | -0.0137 (-0.025, -0.003)* | -0.017 (-0.030, -0.005)* | -0.014 (-0.262, -0.001)* |
| Platelets | -1.370 (-2.721, -0.072) | -1.112 (-2.472, 0.247) | -0.993 (02.452, 0.467) | -1.502 (-3.157, 0.153) |

† adjusted for age, gender, ethnicity (Caucasian vs non Caucasian), clinical diagnosis, number of autoantibodies, concomitant steroid, antimalarial and immunosuppressant use

‡log-transformed values

*p<0.05

Table S4: Linear regression models of log-adjusted NAR expression with autoantibodies

|  | Ds-DNA | Ro | La | Smith | RNP | Chromatin | Any ENA (Ro, La, Smith, RNP) |
| --- | --- | --- | --- | --- | --- | --- | --- |
| TLR7 | 0.107 (-0.081, 0.294) | -0.063 (-0.248, 0.122) | 0.176 (-0.063, 0.416) | 0.329 (0.094, 0.564)* | 0.0496 (-0.149, 0.246) | 0.185 (-0.022, 0.392) | 0.030 (-0.140, 0.202) |
| TLR9 | -0.0367 (-0.196, 0.122) | -0.182 (-0.337, -0.028)* | -0.079 (-0.125, 0.283) | -0.045 (-0.249, 0.159) | -0.012 (-0.181, 0.156) | 0.057 (-0.120, 0.234) | -0.105 (-0.250, 0.039) |
| TLR3 | 0.100 (-0.139, 0.040) | -0.223 (-0.454, 0.008) | 0.0101 (-0.293, 0.315) | 0.268 (-0.033, 0.569) | 0.017 (-0.234, 0.268) | 0.209 (-0.539, 0.471) | -0.097 (-0.314, 0.119) |
| DDX58 | 0.146 (-0.127, 0.419) | 0.342 (0.079, 0.605)* | 0.360 (0.015, 0.705) | 0.688 (0.356, 1.020)* | 0.439 (0.159, 0.719)* | 0.721 (0.440, 1.00)* | 0.493 (0.258, 0.730)* |
| MB21D1 | 0.0184 (-0.138, 0.175) | -0.168 (-0.320, -0.017)* | 0.041 (-0.159, 0.241) | 0.031 (-0.169, 0.231) | -0.016 (-0.181, 0.150) | 0.110 (-0.063, 0.283) | -0.089 (-0.231, 0.052) |
| TMEM173 | 0.031 (-0.115, 0.176) | -0.148 (-0.289, -0.008) | 0.028 (-0.157, 0.214) | 0.052 (-0.137, 0.237) | 0.065 (-0.088, 0.218) | 0.0814 (-0.079, 0.242) | -0.694 (-0.201, 0.02) |

*P<0.05

Figure S1: Distribution of ISG score according to classification criteria for the primary diagnosis

The figure shows the ISG scores according to internationally recognised classification criteria for each patient’s primary diagnosis. The criteria used as highlighted below.

Criteria used:

SLE: Updated 1997 ACR Classification Criteria [1]

Sjogren’s syndrome: ACR/EULAR 2016 Criteria for Primary Sjogren’s Syndrome [2]

Scleroderma: 2013 ACR/EULAR Classification Criteria for Systemic Sclerosis [3]

Myositis: Bohan and Peter 1975 Classification Criteria [4]

Figure S2: Expression of NARs according to disease phenotype

References

1. Hochberg MC. Updating the American College of Rheumatology revised criteria for the classification of systemic lupus erythematosus. Arthritis Rheum. 1985;28:80-6

2. Shiboski et. al. 2016 American College of Rheumatology/European League Against Rheumatism Classification Criteria for Primary Sjogren’s Syndrome: a consensus and data-driven methodology involving three international patient cohorts. Ann Rheum Dis. 2017;76:9-16

3. van den Hoogen et. al. Classification Criteria for Systemic Sclerosis: An ACR-EULAR Collaborative Initiative. Arthritis Rheum. 2013;65:2737-47

4. Bohan A, PeterJB. Polymyositis and dermatomyositis. N Engl J Med. 1975;292:344-7

5. Alarcon-Segovia D, Villareal, M. Classification and diagnostic criteria for mixed connective tissue disease. In: Kasukawa R, Sharp GC, eds. Mixed connective tissue disease and anti-nuclear antibodies. Amsterdam: Elsevier Science. 1987;33–40.
